# Altered Hemorheology in Fontan Patients in Normoxia and After Acute Hypoxic Exercise

**DOI:** 10.3389/fphys.2019.01443

**Published:** 2019-11-22

**Authors:** Julian Alexander Härtel, Nicole Müller, Ulrike Herberg, Johannes Breuer, Daniel Alexander Bizjak, Wilhelm Bloch, Marijke Grau

**Affiliations:** ^1^Department of Molecular and Cellular Sports Medicine, German Sport University Cologne, Cologne, Germany; ^2^Department for Pediatric Cardiology, University Hospital Bonn, Bonn, Germany

**Keywords:** exercise, Fontan circulation, hypoxia, red blood cells, rheology

## Abstract

**Background:**

The Fontan circulation is a unique palliation procedure for several congenital heart defects. Impaired exercise capacity has previously been demonstrated in these patients and also a higher risk for cardiopulmonary mortality. Hemorheology was shown to affect cardiopulmonary capacity and in turn to be affected by regular exercise and hypoxia but none of these have been investigated in Fontan patients so far. The aim of this study was to detect general differences in hemorheology in normoxia as well as possible altered hemorheological responses to hypoxia exposure and hypoxic exercise between Fontan patients and healthy controls.

**Methods and Findings:**

26 Fontan patients and 20 healthy controls performed an acute exercise test (AET) on a bicycle ergometer under hypoxia with ambient 15.2% oxygen saturation (sO_2_). Blood samples were taken at rest in normoxia (T0), at rest in hypoxia (T1), after maximum exhaustion in hypoxia (T2), and after 50 min recovery in normoxia (T3). Hemorheological and blood parameters were investigated. Additionally, arterial stiffness was tested at T0. Red blood cell (RBC) deformability, NOx, erythropoietin (EPO) concentration, RBC count, hemoglobin (Hb) concentration and hematocrit (hct) were significantly increased in Fontan patients compared to controls. Same was observed for arterial stiffness. No changes were observed for RBC aggregation, fibrinogen concentration, free radical levels and vascular endothelial growth factor (VEGF). Hypoxia exposure did not change parameters, whereas exercise in hypoxia increased aggregation and hct significantly in both groups. Fontan patients showed significantly increased aggregation-disaggregation balance compared to controls.

**Conclusion:**

Acute hypoxia exposure and exercise under hypoxia might have similar impact on hemorheology in Fontan patients and controls and was clinically well tolerated. Nevertheless, exercise alters aggregation and possibly hemodynamics which requires special attention in Fontan patients.

## Introduction

The Fontan circulation is the current therapy for patients with severe congenital heart diseases (CHD) leading to a univentricular heart like tricuspid atresia or HLHS. In this unique palliation procedure the inferior and superior *vena cava* are connected to the pulmonary artery resulting in a univentricular circulation with passive pulmonary perfusion called Fontan circulation (Fontan and Baudet, 1971). Especially the blood flow in the pulmonary vessels is of vast research interest, since it is thought to restrict circulatory capacity, because pulmonary blood flow cannot be actively increased due to a lacking subpulmonary pump.

Previous studies examined the cardiopulmonary capacity of Fontan patients, since peak oxygen uptake ($\dot{V}$O_2__peak_) is a quantitative predictor of mortality (Kodama et al., 2009). In Fontan patients, physical capacity is significantly reduced (Driscoll, 1990). Studies reported approximately 60–70% of $\dot{V}$O_2__peak_ and 47–80% of peak workload compared to healthy references (Ohuchi et al., 2001; Takken et al., 2007; Duppen et al., 2015). Reasons for these impairments are under discussion and include impaired cardiac function (e.g., impaired cardiac output), differences in skeletal muscle function, impaired lung function and/or endothelial dysfunction (Larsson et al., 2003; Binotto et al., 2005; Cordina et al., 2013; Lambert et al., 2013; Turquetto et al., 2017, 2018).

With increasing time after completion of the Fontan circulation patients show reduced sO_2_ (Tomkiewicz-Pająk et al., 2013). This, amongst others, might result from postoperative occurrence of veno-venous collaterals, because of increased VEGF (Suda et al., 2004; Tomkiewicz-Pająk et al., 2013). Chronically reduced sO_2_ in turn leads to adaptation processes like increased numbers of RBCs, increased Hb and increased hct which might impact blood viscosity, vascular resistance and RBC properties (e.g., aggregation, deformability) (Connes et al., 2013; Patel et al., 2013; Ycas et al., 2015; Chou et al., 2016). RBC deformability is an important cell property for oxygen and nutrient supply in the microcirculation and depends on several factors of which cellular NO availability is one of them (Bor-Kucukatay et al., 2003; Suhr et al., 2012). Exercise has been shown to positively influence RBC rheology but has also been described to increase free radicals, which might negatively affect RBC rheology (Powers et al., 2011). Such coherencies have not been described for Fontan patients so far.

Morphological alterations of vasculature were previously described in Fontan patients with higher arterial stiffness and increased intima-media-thickness, resulting in shear-stress changes (Lambert et al., 2013; Ridderbos et al., 2015). This might have an impact on hemorheology, even though no differences in RBC aggregation and deformability were demonstrated in 20 Fontan patients compared to ASD patients in a previous study (Cheng et al., 2016).

It has been recognized for years that hemorheological responses are altered under hypoxic conditions in healthy subjects, because of oxygen-dependent regulatory mechanisms of RBC properties (Mao et al., 2011; Chou et al., 2016; Grau et al., 2016). This might impair oxygen supply within the microcirculation and thus be relevant during altitude exposure and, given the changes described above, especially for Fontan patients. Nevertheless, influence of hypoxia on RBC deformability is still controversial in literature. Previous studies demonstrated a decrease or no change of deformability under hypoxic conditions (Tuvia et al., 1992; Kaniewski et al., 1994; Parthasarathi and Lipowsky, 1999; Moon et al., 2016), while others report an increase (Wei et al., 2016).

Also, increased NO levels due to hypoxia have previously been shown, which could affect deformability. Gladwin and Kim-Shapiro (2008) demonstrated increased NO in a hypoxic environment, regulating vasodilation.

Exercise during short-time hypoxia exposure was shown to be clinically well tolerated in Fontan patients (Staempfli et al., 2016; Naqvi et al., 2018) but still, hypoxia exposure during leisure activities like hiking or even during flights, where inner cabin pressure corresponds to altitudes of 1,800–2,500 m asl, might thus bear a health risk for Fontan patients and requires investigations. Also, effects of hypoxia and exercise during hypoxia on hematological and hemorheological parameters have not been investigated in Fontan patients and would add important information on RBC regulation in Fontan patients. Furthermore, general differences in hematological and hemorheological parameters between Fontan patients and healthy controls are presented.

## Materials and Methods

### Study Subjects

The 26 Fontan patients and 20 healthy, untrained, age-, sex-, and BMI-matched controls were included in the study. Anthropometric data, medication and underlying cardiac defect are described in Table 1.

**TABLE 1:** Participant’s characteristics.

		**Fontan (*n* = 26)**	**Control (*n* = 20)**	**Significance (*p*-value)**
Age (years)		17.5 [10, 34]^∗^	18 [14, 33]^∗^	ns (0.67)
Gender (f/m)		10/16	9/11	
Body mass index (kg/m^2^)		22.4 ± 4.4	22.26 ± 3.32	ns (0.89)
Body surface area (Mosteller) (m^2^)		1.67 ± 0.3	1.77 ± 0.23	ns (0.24)
Type of Fontan circulation				
	I. Fenestration	2		
	II. Aortic arch reconstruction with patch	7		
Underlying cardiac defect				
	*I. Functional left ventricle*			
	Tricuspid atresia	4		
	Double inlet left ventricle (DILV)	7		
	Single inlet left ventricle	1		
	Single ventricle with ccTGA	1		
	*II. Functional right ventricle*			
	Hypoplastic left heart syndrome	7		
	Double outlet right ventricle (DORV)	2		
	Single ventricle with ccTGA	4		
Medication				
	I. Cardioselective β-Blocker	7		
	II. ACE-inhibitors	11		

*^∗^Median [min, max], ns = not significant.*

Patients were only included when they showed a good current health status (NYHA class I or II) and mental and physical ability to absolve a cycle ergometry in sitting position.

Fontan patients with failing Fontan, mental disability, $\dot{V}$O_2__peak_ < 45% of predicted values, age <10 years and/or pregnancy were excluded from the study.

Additional exclusion criteria for controls were cardiovascular disorders, smoking and/or athlete status.

The study protocol was approved by the ethics committee of the University of Bonn (application number 335/2014) and corresponded with the Declaration of Helsinki. All participants/parents gave written informed consent to participate in this study.

### Study Protocol

**FIGURE 1:**
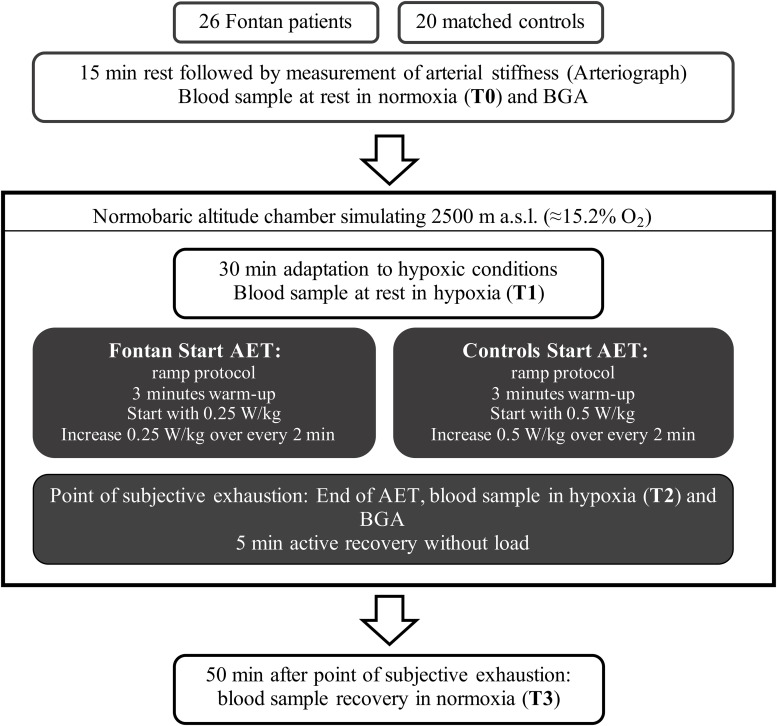
Study protocol. asl = above sea level, AET = acute exercise testing, BGA = blood gas analysis.

Figure 1 presents the detailed study procedure. Briefly, in this explorative study 46 participants (26 Fontan patients, 20 controls) completed an exercise test on a sitting bicycle ergometer under hypoxic conditions. Blood samples of all participants were taken at four different timepoints: T0: normoxia, T1: hypoxia, T2: hypoxia and exercise and T3: after 50 min of recovery in normoxia. All parameters were examined at these timepoints unless otherwise described. Blood samples were examined on (1) blood count (T0), (2) EPO (T0, T1, T2), (3) RBC aggregation, (4) RBC deformability, (5) RBC NOx, (6) plasma fibrinogen (T0, T2), (7) free reactive oxygen/nitrogen (ROS/RNS) species (T0) and (8) VEGF (T0). Moreover, BGA was performed in normoxia (T0) and under hypoxic conditions after exercise (T2). Systemic arterial stiffness was measured non-invasively after 15 min resting, before entering the altitude chamber (T0).

### Acute Exercise Test in Normobaric Hypoxia

All participants absolved a cycle ergometry test in a normobaric altitude chamber (Höhenbalance, Cologne, Germany). Ambient oxygen was reduced by membrane processes and was replaced by nitrogen at constant air pressure (≈1,013 hPa) to 15.2% oxygen and 84.8% nitrogen, corresponding to a simulated altitude of 2,500m asl. AET was done using a computer controlled sitting bicycle ergometer (ERG 911 Plus, Ergosana GmbH Schiller, Bitz, Germany) with Custo Diagnostics software (Custo Diagnostics, V. 5.4, Custo med GmbH, Ottobrunn, Germany). A 12-lead ECG was continuously recorded (Custo Diagnostics, V. 5.4, Custo med GmbH, Ottobrunn, Germany) and systolic (RR_sys_) and diastolic (RR_dia_) blood pressure was measured every 2 min (Tango M2, SunTech Medical Inc., Morrisville, NC, United States). A finger pulse oximeter was used for observation of transcutaneous sO_2_ (V100, GE Healthcare, Chicago, IL, United States).

Two different load protocols were used for either Fontan patients or controls to achieve loading periods of 8–15 min (Figure 1). Endpoint of ergometry was marked by subjective exhaustion (BORG-Scale ≥16, cadence ≥60 rpm could not be sustained) and was followed by 5 min of recovery. Before exercise in normoxia (T0) and immediately after maximal exhaustion in hypoxia (T2) venous blood was sampled for BGA (RAPIDLab1200, Siemens Healthineers, Erlangen, Germany).

### Arterial Stiffness

Arterial stiffness was measured at T0 using the Arteriograph (TensioMed Kft., Budapest, Hungary). This device allows the non-invasive measurement of PWV and AIx by analysis of oscillometric captured pressure curves. The underlying technique of this device is based on plethysmography: A cuff is placed at the upper arm and initially measures blood pressure. Then, the cuff is filled again with a pressure of 35 mmHG above RR_sys_. Because the tissue of the upper arm is barely compressible, every change of the arterial pressure is followed by pressure changes (oscillations) in the inflated cuff. This can be detected by the Arteriograph and is visualized as pulse waves by the Arteriograph software (V. 3.0.0.4, TensioMed Kft., Budapest, Hungary). The software then calculates PWV (time between beginning of first and beginning of second pulse wave based on distance between jugulum and symphysis) and AIx (underlying formula: $\frac{P\,2-P\,1}{P\,P}$, with *P*1 = amplitude of the first wave, *P*2 = amplitude of the second wave and *PP* = pulse pressure). Reliability was validated in several studies (Baulmann et al., 2008; Horvath et al., 2010).

Before measurement, study subjects were not allowed to take stimulating substances (e.g., coffee) and had to lie on a bench for 15 min in silence to reduce sympathetic activity. To exclude influences of drugs, the Fontan group was subdivided in Fontan patients (1) taking ß-blockers, (2) taking ACE-antagonists, (3) with no medication. Results were compared.

### Blood Sampling and Processing

Venous blood was sampled from the cephalic vein using a peripheral venous catheter (B. Braun Melsungen AG, Melsungen, Germany) and anticoagulated using sodium heparin vacutainer for the above described study parameters or EDTA vacutainer (Becton, Dickinson and Company, Franklin Lakes, NJ, United States) for blood count by the central laboratory of the University Hospital Bonn. Immediately after sampling, whole blood was transferred into a clean tube and further processed for aggregation measurements (see below). Another aliquot (10 μl) was used for RBC deformability measurements (see below). Remaining blood was centrifuged at 3,600 × *g* for 2 min and 4°C and plasma supernatant was transferred into clean tubes, snap frozen and stored at −80°C for measurements of EPO and Fibrinogen. One aliquot of RBC pellet was mixed with 0.1 mol phosphate buffered saline (PBS; pH 7.4) to obtain a 2 × 10^7^ cells/ml suspension, snap frozen and stored at −80°C until measurements. Another aliquot was mixed with a nitrite preservation solution, snap frozen and stored at −80°C for RBC NOx measurements (see below). EPO, Fibrinogen and VEGF were measured using human enzyme-linked immunosorbent assays (ELISA) (Human EPO ELISA, Human VEGF ELISA, R&D Systems, Minneapolis, MN, United States; Human Fibrinogen ELISA, Abcam, Cambridge, United Kingdom). Free ROS/RNS were measured using the OxiSelect *in vitro* ROS/RNS Assay Kit (Cell Biolabs Inc., United States).

### RBC NOx

Concentrations of RBC NOx were measured according to published protocols (Pelletier et al., 2006; Hendgen-Cotta et al., 2008).

To preserve nitrite in RBC, a ferricyanide-based preservation solution was added to the RBC in a 1:5 ratio. The solution consists of 0.8 M ferricyanide, 0.1 M N-ethylmaleimide, and IGEPAL (10% of the total volume of preservation solution). To remove proteins, RBC samples were mixed in a 1:2 ratio with methanol (VWR International, Radnor, United States) and centrifuged at 21,000 × *g* for 15 min at 4°C. The detailed method of sample measurement was previously described (Grau et al., 2007).

Briefly, sample supernatants were transferred into clean tubes. To reduce nitrite to NO gas, the supernatants were then injected into an acidified tri-iodide solution. Transferred by a helium-gas-stream, the resulting NO was purged into an ozone-based chemiluminescence NO detector (CLD 88e, EcoPhysics AG, Duernten, Switzerland). There, NO reacted with ozone to an excited state of nitrogen dioxide, emitting light, which then could be detected by the CLD. Using calibration solutions with known nitrite concentrations allowed to calculate the sample nitrite content with the Chart FIA software (EcoPhysics AG, Duernten, Switzerland) by integrating the area under the curve. Every blood sample was measured three times and nitrite concentrations were corrected for nitrite concentration of methanol and preservation solution.

### RBC Rheology

Both, RBC deformability and aggregation were measured in anticoagulated whole blood by a LORRCA (RR Mechatronics, Hoorn, Netherlands) (Hardeman et al., 2001).

For measurement of deformability, RBC were mixed in a 1:250 ratio with a viscous polyvinylpyrrolidone solution (PVP, viscosity: 29 cP; RR Mechatronics, Hoorn, Netherlands). The RBC/PVP samples were exposed to nine increasing shear stresses between 0.3 and 50 Pa and investigated with a laser beam directed through the samples. The LORRCA software analyzed the diffraction patterns, produced by the deformation changes of the RBC and calculated an elongation index (EI) for each shear stress. The underlying equation for calculation of the EI was: EI = (A − B)/(A + B). Thereby, “A” represents the length of the major axis and “B” represents the length of the minor axis of the RBC. All values were measured twice, and means were calculated.

Before RBC aggregation measurement, all samples were fully oxygenated for 15 min with the use of a Roller Mixer (36 rpm; RM5, Karl Hecht KG, Germany). Native Hct was used to detect *in vivo* differences between groups. This was not according to the common experimental procedure, where aggregation is investigated with standardized hct, because hct might influence aggregation. Nevertheless, *in vivo* measurements were done to explain possible alterations in rheology during exercise with possible changes in hct and thereby to give better recommendations for Fontan patients, since they have to deal with higher hct during hypoxic exercise. Samples were measured at 37°C and investigated by syllectometry. Thereby, initially shear-stress exposed RBC were investigated by a laser beam. Changes of backscattered light were recorded over 120 s using two photodiodes and presented as a graph (syllectogram). The syllectogram was evaluated by distinguishing four different stages: (1) disaggregation stage, which is achieved by increasing shear rate to 500 s^–1^ for 3 s. (2) shape recovery stage, after the stop of motor. (3) rouleaux formation stage in 2D directly followed by (4) 3D aggregation stage. The LORRCA software calculated the AI and aggregation half time (*t*_1__/__2_), which is defined by time required for half maximum change in aggregation signal (Tripette et al., 2009). To demonstrate the threshold shear rate balancing RBC aggregation and disaggregation, an iteration procedure was accomplished to primarily calculate dIsc min. This parameter defines the minimum change in backscatter intensity during the iteration procedure, representing the shear rate, where RBC start to disaggregate [*y* at dIsc min (s^–1^)].

### Statistical Analysis

GraphPad Prism (V. 7.0e, GraphPad Software, San Diego, CA, United States) was used for statistical analysis. Results were tested for normal distribution using D’Agostino–Pearson normality test. To analyze changes in parameters at the four different sample timepoints within groups repeated-measures ANOVA and Tukey’s multiple comparison *post hoc* test was used for parameters fitting a normal distribution. Otherwise Friedman test and Dunn’s multiple comparison test was applied for variables not fitting a normal distribution.

Pre- and post-exercise values (T0 vs. T2) within groups were compared using student’s paired *t*-tests.

Because of different group sizes, student’s unpaired *t*-test was used to test group differences at similar timepoints. Group sizes varied because (a) sample volume was insufficient to measure all parameters or (b) errors during measurement. Mann–Whitney *U* test was applied for variables not fitting a normal distribution.

Correlations between different parameters were determined using univariate linear regression and Pearson’s correlation coefficient.

Quantitative values are given in mean ± standard deviation (SD) unless otherwise described.

*p*-values ≤ 0.05 are classified statistically significant. Different significance levels in figures are presented as ^∗^, using the following convention: ^∗^*p* ≤ 0.05, ^∗∗^*p* ≤ 0.01, ^∗∗∗^*p* ≤ 0.001, ^****^p ≤ 0.0001, ns = not significant.

## Results

### AET Parameters and BGA Significantly Differed Between Groups

During ergometry, vital parameters and exercise parameters were recorded constantly to control the medical situation. Peak workload expressed as power per weight (W/kg) was significantly impaired in Fontan patients (Table 2). SpO_2_ significantly decreased in the Fontan group and control group from baseline in normoxia (T0) to baseline in hypoxia (T1) (Fontan and Control *p* ≤ 0.0001) and again during the AET at the point of maximum exhaustion (T2) (Fontan and Control *p* ≤ 0.0001). At rest in normoxia and hypoxia (T0 and T1) as well as at peak workload (T2), Fontan patients had significantly lower SpO_2_ compared to controls (Tables 2, 3), but the loss of SpO_2_ at maximum exhaustion was similar. A statistically significant correlation between SpO_2_ and peak workload could not be detected (data not shown).

**TABLE 2:** Results of **(A)** AET and **(B)** BGA and fibrinogen, *n* = number of values, ns = not significant.

**(A)**	**REST_hypoxia_ (T1)**			**PEAK_hypoxia_ (T2)**		
	**Fontan (*n* = 26)**	**Control (*n* = 20)**	**Significance (*p*-value)**	**Fontan (*n* = 26)**	**Control (*n* = 20)**	**Significance (*p*-value)**
Workload (W/kg)				1.69 ± 0.29	2.97 ± 0.61	≤0.0001
SpO_2_ (%)	87 ± 5	94 ± 2	≤0.0001	83 ± 6	90 ± 4	≤0.0001
RR_sys_ (mmHg)	113 ± 15	123 ± 13	0.03	149 ± 26	176 ± 28	0.002
RR_dia_ (mmHg)	73 ± 12	73 ± 9	ns	73 ± 13	72 ± 18	ns
EPO (mlU/ml)	11.73 ± 9.8	5.76 ± 4.15	0.0055	12.81 ± 10.99	5.52 ± 5.02	0.0101
	(*n* = 21)	(*n* = 20)		(*n* = 21)	(*n* = 20)	

**(B)**	**REST_normoxia_ (T0)**			**PEAK_hypoxia_ (T2)**		
	**Fontan (*n* = 21)**	**Control (*n* = 20)**	**Significance (*p*-value)**	**Fontan (*n* = 21)**	**Control (*n* = 20)**	**Significance (*p*-value)**

Hematocrit (%)	46.5 ± 4.7	41.2 ± 3.8	0.0009	48.25 ± 6.92	45.56 ± 4.3	ns
	(*n* = 19)	(*n* = 17)		(*n* = 16)	(*n* = 18)	
Venous pH	7.41 ± 0.03	7.4 ± 0.03	ns	7.34 ± 0.04	7.26 ± 0.05	≤0.0001
Venous lactate (mmol/l)	1.48 ± 0.3	1.57 ± 0.53	ns	5.09 ± 1.66	8.97 ± 2.01	≤0.0001
				(*n* = 19)	(*n* = 17)	
Base excess (mmol/l)	−1.52 ± 1.27	−0.07 ± 1.57	0.002	−5.87 ± 2.16	−8.49 ± 2.69	0.002
HCO_3_^–^ (mmol/l)	22.68 ± 1.67	24.91 ± 2.2	0.0007	19.52 ± 3.24	18.16 ± 2.87	ns
Fibrinogen (mg/ml)	2.88 ± 0.66	2.78 ± 1.85	ns	3.94 ± 1.48	3.94 ± 1.7	ns
	(*n* = 20)			(*n* = 20)		

**p*-values refer to the group comparison of Fontan patients and controls.*

**TABLE 3:** Blood count, SpO_2_, EPO, ROS/RNS, and VEGF at T0 in Fontan patients and controls.

	**Fontan**	**Control**	**Significance**
	**(*n* = 21)**	**(*n* = 20)**	**(*p*-value)**
SpO_2_ (%)	92 ± 4.1	99 ± 1	≤0.0001
EPO (mlU/ml)	12.77 ± 10.55	5.33 ± 4.33	0.0057
Number of erythrocytes (×10^12^/l)	5.21 ± 0.43	4.89 ± 0.43	0.0187
Hemoglobin (g/dl)	15.55 ± 1.51	14.11 ± 0.98	0.0009
Hematocrit (%)	44.81 ± 3.44	41.7 ± 2.62	0.0024
Red blood cell distribution width (%)	13.79 ± 1.3	12.79 ± 0.97	0.0035
MCV (fl)	82.38 ± 11.68	82.6 ± 11.59	ns
MCH (pg)	29.9 ± 1.73	29.1 ± 1.65	ns
MCHC (g/dl)	34.62 ± 1.28	34.2 ± 2.07	ns
VEGF (pg/ml)	26.5 ± 24.6	25.0 ± 22.2	ns
	(*n* = 26)	(*n* = 19)	ns
ROS/RNS (μmol/l)	71.6 ± 18.1	79.6 ± 14.1	ns
	(*n* = 16)	(*n* = 13)	ns

*ns = not significant.*

In both groups RR_sys_ increased significantly during exercise (Fontan and Control *p* ≤ 0.0001). At rest as well as at maximum exhaustion RR_sys_ was significantly lower in Fontan patients than controls, albeit RR_dia_ developed similar. A correlation between age and RR_sys_ was not seen in any group.

BGA was measured in 21 Fontan patients and 20 controls. Results are presented in Table 2. Hct was significantly higher in Fontan patients at rest. At rest, base excess and bicarbonate was significantly different between the groups but all means stayed in physiological range. Controls showed greater decreases in venous pH and base excess and had also significant higher venous lactate concentrations at peak workload, even though lactate concentration significantly increased in both groups (Fontan and Control *p* ≤ 0.0001). Hct values significantly increased in both groups (Fontan *p* = 0.0063, Control *p* = 0.0081).

### Arterial Stiffness Was Significantly Higher in Fontan Patients

**FIGURE 2:**
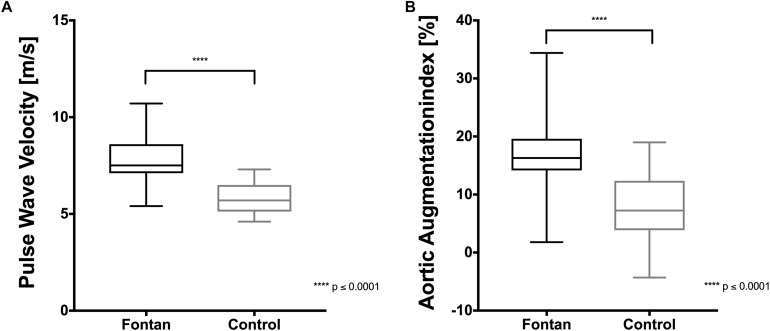
Arterial stiffness in Fontan patients and healthy controls. **(A)** Pulse wave velocity (PWV) and **(B)** aortic augmentation index (AIx) were significantly higher in Fontan patients compared to healthy control.

Aortic PWV and AIx were measured in 19 Fontan patients and 20 controls. Results of PWV and AIx are presented in Figure 2. In both parameters, mean of Fontan patients was significantly higher in contrast to controls. When comparing subgroups of Fontan patients taking different medication (ß-blockers, ACE-antagonists), no significant differences in PWV and AIx were found in comparison to Fontan patients without medication, but they also showed higher values in comparison to controls. Patch implantation for building a neo-aorta like in patients with HLHS did not show differences to those without patches (data not shown). A statistically significant correlation between AIx and RR_sys_ could not be seen.

### Altered Blood RBC Parameters in Fontan Patients

Blood count parameters of 21 Fontan patients and 20 controls were measured at rest in normoxia. EPO was measured at rest in normoxia (T0), after 30 min of hypoxia exposure (T1) and at maximum exhaustion (T2). In addition, VEGF was measured in 20 Fontan patients and 19 controls at rest in normoxia. Results are summarized in Table 3.

Fontan patients showed significant higher EPO levels, higher numbers of RBC, higher Hb concentrations and higher hct levels compared to controls. Also, RDW was significantly increased. 24% of Fontan patients exceeded the reference value (>14.5%) in contrast to 5% of controls. RDW negatively correlated with MCH in the Fontan group, but not in controls (*p* = 0.018, Pearson *r* = −0.51). Fontan patients showed a trend of an inverse correlation between RDW and deformability, but this was not significant (*p* = 0.077, Pearson *r* = −0.39), nor was it in the control group. RBC properties including MCV, MCH and MCHC were similar between the groups. Neither short time altitude exposure (≈2 h), nor maximum physical activity in hypoxia affected EPO concentrations in any group (Table 2a). VEGF stayed in physiological ranges in Fontan patients and did not show significant differences to controls (Table 3). Only one patient showed a higher value compared to a peak reference value of 55 pg/ml, but also two controls did.

### RBC Deformability Was Higher in Fontan Patients

**FIGURE 3:**
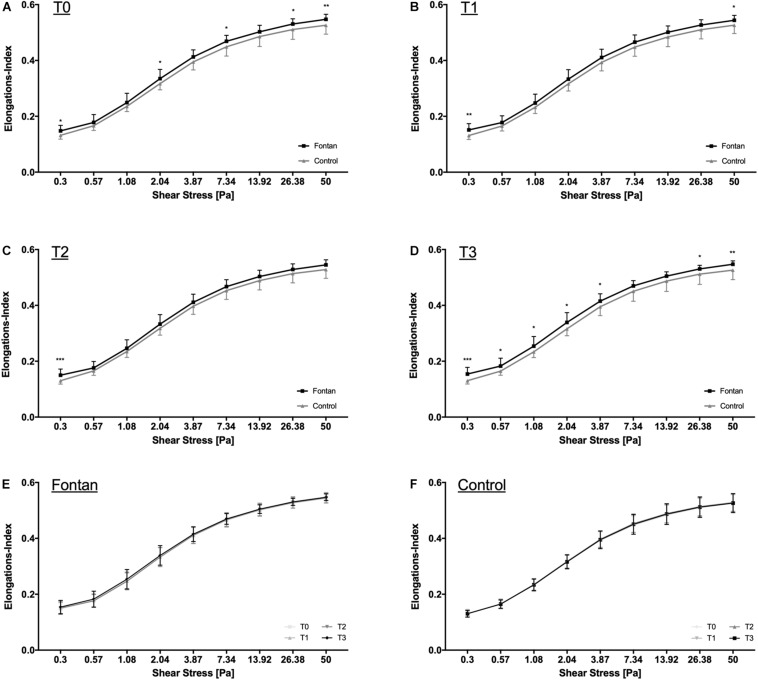
RBC deformability measurements. RBC deformability for **(A)** T0, **(B)** T1, **(C)** T2, and **(D)** T3 was significantly higher in Fontan patients compared to healthy controls. Within group comparison of RBC deformability revealed no change in deformability during intervention for **(E)** Fontan patients and **(F)** healthy controls.

Deformability was measured in 26 Fontan patients and 20 controls. Figures 3A–D suggests that RBC deformability was significantly higher in Fontan patients at all four measured timepoints.

Figures 3E,F indicates that none of the two interventions (hypoxia exposure and physical activity) had an impact on RBC-deformability.

### RBC NOx Increased in Fontan Patients

**FIGURE 4:**
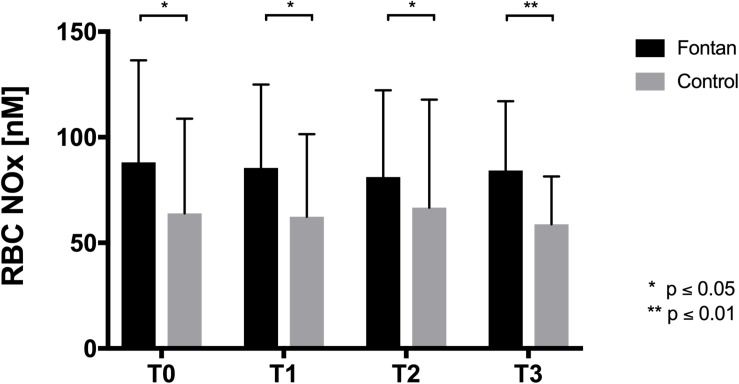
RBC NOx concentration RBC of Fontan patients showed significantly higher NOx levels compared to healthy controls at all timepoints, but NOx concentrations remained unaltered during intervention.

Blood samples from 26 Fontan patients and 20 controls were tested for RBC NOx concentration. At all measured timepoints RBC NOx levels were significantly higher in the Fontan group (Figure 4). Hypoxia exposure (T1) and sports intervention in hypoxia (T2) did not affect NOx concentrations.

### Oxidative Stress in RBC Comparable Between Groups

Concentration of ROS/RNS was measured in 16 Fontan patients and 13 controls in normoxia (T0). Results are presented in Table 3. Differences were not found between groups.

### Aggregation-Disaggregation Balance Was Increased in Fontan Patients After Exercise Intervention in Hypoxia

**FIGURE 5:**
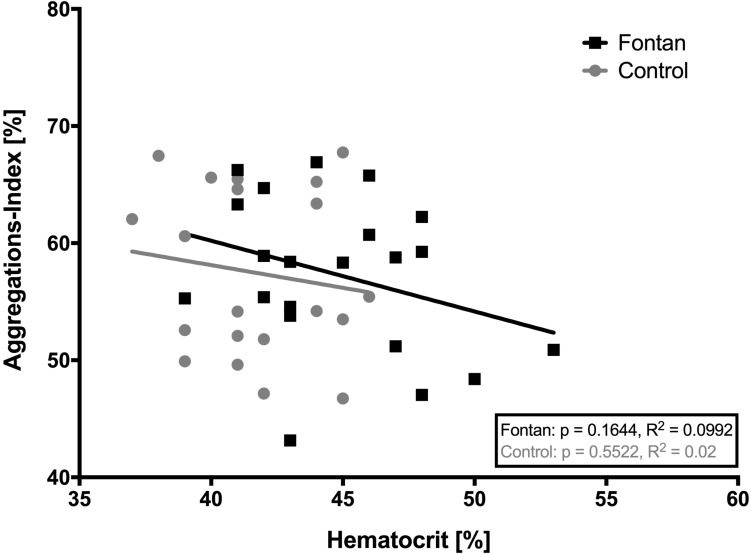
Correlation of native hematocrit (hct) with aggregations-index (AI). No significant correlation was found in any group. This might indicate that AI in this study was not influenced by native hct.

**FIGURE 6:**
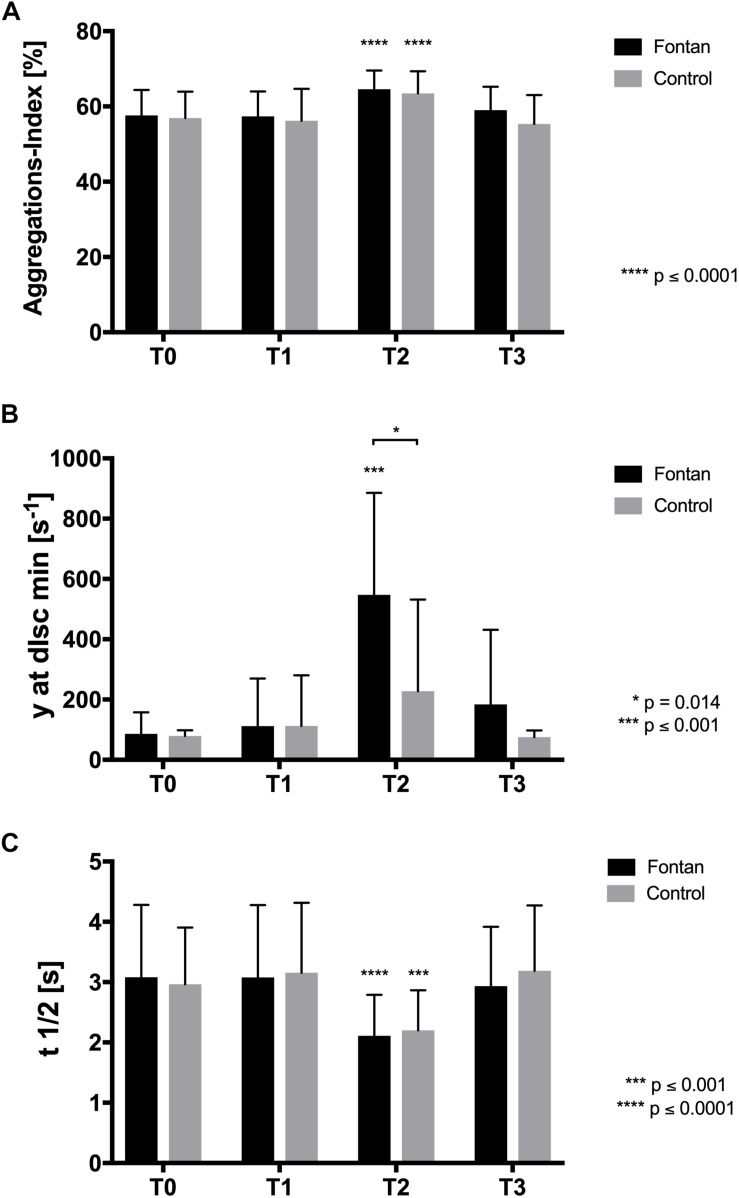
Aggregation and disaggregation balance. **(A)** Aggregations index (AI) was comparable between Fontan patients and healthy controls. Values significantly increased at T2 in both groups and returned to initial value at T3. **(B)** *y* at dIsc min representing the minimum shear stress balancing RBC aggregation-disaggregation was comparable between Fontan patients and healthy controls at T0 and T1, respectively. At T2, *y* at dIsc min significantly increased in Fontan patients compared to control. **(C)** Aggregation half-time (*t*_1/2_) was comparable between the two tested groups. Values significantly decreased in both groups at T2.

**FIGURE 7:**
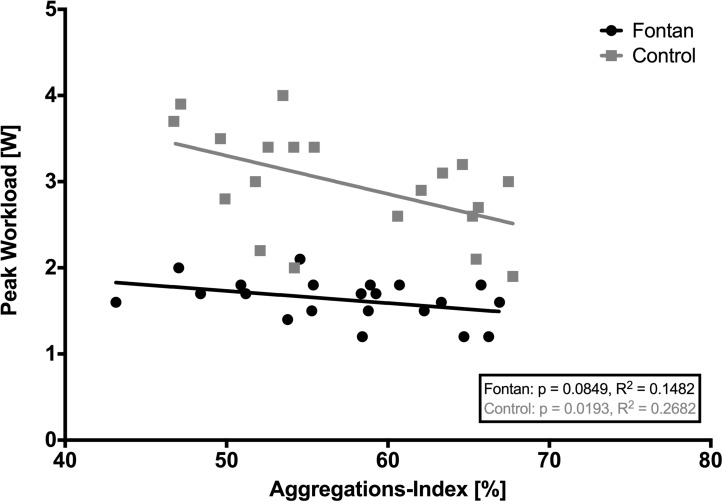
Correlation of aggregations-index (AI) and peak workload. A statistically significant correlation between AI and peak workload could be demonstrated in the control group. Possibly this correlation could not be seen in the Fontan group, because Fontan patients might not have reached their individual maximum physical capacity.

Because AI measurements are normally performed with standardized hct, correlation analysis of hct and AI were performed. Figure 5 demonstrates that there was no significant correlation between hct and AI. AI and *t*_1__/__2_ is presented at four different timepoints in Figures 6A,C. Values did not differ between Fontan patients and controls at any of the measuring points. Nevertheless, exercise in hypoxia (T2) led to significant changes in AI and *t*_1__/__2_ in both groups: AI increased, while *t*_1__/__2_ was correspondingly lower. A significant correlation of AI and peak workload could be demonstrated in the control group, but not in the Fontan group (Figure 7). The correlation of venous peak lactate and AI was not significant in any group.

Shear stress used for minimum disaggregation (*y* at dIsc min) was comparable between groups at T0 and increased in both groups after exercise intervention (T2). But only Fontan patients showed a significant increase and significant higher values compared to controls (Figure 6B).

### Plasma Fibrinogen

Plasma fibrinogen was measured in 20 Fontan patients and 20 controls. Values at T0 and T2 are presented in Table 2. In both groups fibrinogen was significantly increased after exercise in hypoxia (Fontan *p* = 0.0049, Control *p* = 0.04), but did not differ between both groups. A statistically significant correlation between AI and plasma fibrinogen concentration was only found for Fontan patients at T0 (*p* = 0.013, Pearson *r* = 0.55, data not shown).

## Discussion

This is the first study demonstrating responses of rheological parameters in Fontan patients on hypoxic conditions and exercise in hypoxia. Moreover, basic differences between Fontan patients and matched controls of hemorheology and vascular parameters are presented.

### General Differences Between Fontan Patients and Controls

With approximately 57% of maximum workload of controls this study showed significantly reduced exercise capacity in Fontan patients in hypoxia. Under altitude conditions partial oxygen pressure is reduced and results in immediate increases of the ventilation or the heart rate. Also, $\dot{V}$O_2__peak_ and heart rate reserve and thus performance capacity are reduced under hypoxic conditions (Mairbäurl, 2000).

The results of the present study indicate reduced performance capacity of Fontan patients in general, but a similar decrease during hypoxia in both Fontan patients and controls, since capacities of 47–80% compared to controls were reported in normoxia (Ohuchi et al., 2001; Takken et al., 2007). Therefore, hypoxia until 2,500 m asl might not have greater impact on peak workloads of Fontan patients than on controls, even though performance capacity in normoxia was not tested in these patients what should be done in future studies. This finding is also underlined by herein presented SpO_2_-values, because the relative and absolute decrease at T2 was similar to controls. Furthermore, these results correspond to results of Staempfli et al. (2016), who also investigated exercise performances in 16 Fontan patients and 14 matched controls in hypoxia (3,454 m asl). They demonstrated similar relative maximum workloads in hypoxia with approximately 59% of controls.

Interestingly, increased VEGF levels could not be found in the Fontan group on the basis of reduced SpO_2_ as previously reported (Suda et al., 2004). This might be explained by selection bias in Fontan patients, because only patients with a good current health status were allowed to participate in the present study. Unfortunately, vascular collaterals, which are associated with increased VEGF levels, were not investigated in these patients. Nevertheless, Levy et al. (2004) demonstrated low VEGF expression in Fontan patients with a good surgical result. Furthermore, under the condition of significantly decreased SpO_2_, initial BGA values (pH, lactate, BE, HCO_3_^–^) at rest in Fontan patients and controls were similar, indicating that metabolism of Fontan patients was adapted to decreased sO_2_ and hypoxia exposure might lead to similar effects in BGA parameters.

As a possible compensatory mechanism on the reduced sO_2_, the present data suggest higher EPO levels in Fontan patients followed by higher numbers of RBC and increased hct, which is in concordance to findings of Tomkiewicz-Pajak et al. (2014). The stimulation of erythropoiesis by higher EPO levels might result in a younger and more flexible RBC population (Scholz et al., 1990; Smith et al., 1999; Klipp et al., 2007) but might also stimulate NO production in the endothelium (Cokic et al., 2014) which might positively influence vascular function. NO was also shown to positively influence RBC deformability (Bor-Kucukatay et al., 2003; Suhr et al., 2009). NO is enzymatically produced by the eNOS (endothelial cells) and RBC nitric oxide synthase (RBC-NOS, erythrocytes). At increasing shear stress eNOS and RBC-NOS are activated and produce NO in the vasculature and in RBC (Ulker et al., 2011), which leads to vasodilation and might also result in higher deformability (Bor-Kucukatay et al., 2003; Suhr et al., 2009; Connes et al., 2013). This study demonstrated higher deformability and higher RBC NO values in Fontan patients at all timepoints. Therefore, factors having impact on shear stress might be different in Fontan patients. Higher arterial stiffness, which might be accompanied by increased wall shear stress might thereby influence hemorheology, as increased aortic PWV and AIx were shown in this study in Fontan patients. Increased AIx measured by carotid tonometry was also seen in a study of Lambert et al. in these patients (Lambert et al., 2013). Arterial stiffness was shown to be correlated with higher RR_sys_ (AlGhatrif and Lakatta, 2015). Surprisingly, RR_sys_ was significantly lower in Fontan patients, what may indicate that higher arterial stiffness is a mechanism to compensate reduced cardiac output in these patients and to maintain physiological pressure by increased sympathetic activity, as previously described (Lambert et al., 2013).

Hemorheology is also affected by RBC aggregation. Although the precise mechanism of RBC aggregation is still not explored, several factors, including fibrinogen or RBC cell age have been described to affect RBC aggregation: Previous studies demonstrated increased RBC aggregation due to higher concentrations of fibrinogen (Varlet-Marie et al., 2003; Simmonds et al., 2013). The results presented here did not show differences in aggregation between Fontan patients and healthy controls, which is in concordance to previous findings of Cheng et al. (2016). Also, fibrinogen levels were comparable between the groups.

Just like RBC deformability, RBC aggregation was shown to correlate with cell age. The first report on the effects of erythrocyte age on aggregation behavior appears to be that by Nordt et al., showing that aggregation is higher in old compared to young RBC (Nordt, 1983).

Aggregation values of the present study were measured at native hct to relate possible changes in aggregation to changes in hct. The tested Fontan patients showed higher hct values compared to controls at rest, but this did not result in higher AI. Also, we could not find a statistically significant correlation of hct and AI, as previously reported (Connes et al., 2013), which might suggest that AI might be less dependent on hct in this study. Furthermore, it might be suggested that the RBC tend to show less aggregability, possibly because of younger mean RBC age due to higher EPO levels, as discussed before.

Furthermore, RBC deformability and aggregation are affected by blood parameters including MCV, MCH or MCHC. The results presented herein did not show differences in these blood parameters between Fontan patients and controls neither did the values exceed the normal range. It is thus unlikely that the observed changes were associated to a volume-to-surface shift or increased intra-erythrocytic viscosity.

Even though we could not find a significant correlation of RDW and deformability, as described by Patel et al. (2013), there was a significant difference between groups in RDW, a parameter defining the degree of anisocytosis. Approximately 24% of Fontan patients in this study reached pathologic values (≥14.5%), which might be caused by disturbed erythropoiesis, shortened life span or early reticulocyte release (Said et al., 2017). This result is in agreement with previous findings (Tomkiewicz-Pajak et al., 2014). Reduced RBC lifespan has been shown to be caused by high concentrations of free radicals (Kumar and Rizvi, 2014): Old RBC lose antioxidant capacity and show higher ROS levels (Lutz and Bogdanova, 2013; Kumar and Rizvi, 2014). Here, ROS/RNS levels at rest were similar between Fontan patients and controls suggesting that hemorheological changes observed in this study were not likely related to free radical levels. RDW was shown to correlate negatively with SaO_2_ and MCH (Tomkiewicz-Pajak et al., 2014). In this study we could only find a significant inverse correlation between RDW and MCH in the Fontan group. This might indicate inadequate erythropoiesis possibly because of early iron deficiency in patients with pathologic RDW, as reported by Martinez-Quintana and Rodriguez-González or because of chronically lower oxygen saturations (Martinez-Quintana and Rodriguez-Gonzalez, 2013; Tomkiewicz-Pajak et al., 2014). Nevertheless, iron-deficiency without anemia has previously been demonstrated to have no significant impact on physical capacity and might thereby not affect physical capacity in Fontan patients, neither (Houston et al., 2018).

### Effects of Hypoxia and Exercise on Hemorheological Parameters

Exposure time in hypoxia and oxygen content affect EPO release (Ploszczyca et al., 2018). Thus, it is not surprising, that in this study hypoxia exposure and exercise in hypoxia did not affect EPO concentrations in any of the groups, because total exposure time and altitude was too short (90 min) and too low (2,500 m asl). However, BGA parameters like hct, venous lactate and fibrinogen significantly increased in both groups after exercise in hypoxia. These changes are mainly attributed to the AET and were discussed to result from “hemoconcentration” due to fluid shift, water loss (sweating) and water trapping in muscles (Brun et al., 1998). Interestingly, hct did not significantly differ between Fontan patients and controls after exercise (T2) even though mean hct of Fontan patients was higher. We suggest that Fontan patients did not attain their individual maximum exercise capacity during the AET, because peak lactate concentration at T2 was on a low level and was also significantly reduced compared to controls. Therefore, effects seen in BGA might be greater in controls, but trends were similar. Consequently, Fontan patients are suggested to have even higher hct values after maximum exhaustion.

Results of previous studies describe inconsistent RBC aggregation responses upon exercise (Connes et al., 2013). Aggregation did not differ between the groups during the intervention. Though, aggregation increased at T2 in both groups. The presented results did not show differences in fibrinogen concentration between the groups but did show increased plasma fibrinogen levels at T2. It is thus indicated that higher aggregation observed at T2 might also be related to higher fibrinogen levels caused by hemoconcentration after exercise.

Brun et al. discussed a relationship between physical capacity and RBC aggregation. They demonstrated an inverse correlation of AI and rise in lactate as well as AI and $\dot{V}$O_2__peak_ (Brun et al., 1994, 1995). This study demonstrated a significant correlation of AI (T0) with peak workload in controls, but not in Fontan patients, which could be due to the fact that Fontan patients did not reach their individual maximum exercise capacity, as already discussed. A higher baseline-aggregation might lead to impairments in microcirculation and oxygen supply in the musculature (Vicaut et al., 1994), resulting in lower maximum exercise capacity and lower peak workload. This hypothesis is underlined by a study of Dintenfass et al., showing that fitter patients had lower RBC baseline-aggregation and might also be valid for Fontan patients, since no differences in AI were found compared to controls (Dintenfass and Lake, 1977). Nevertheless, the question if physical fitness influences rheology or vice versa rheology influences physical fitness, cannot be answered, yet.

Interestingly, minimum shear rate to balance RBC aggregation and RBC disaggregation increased after exercise with significantly higher shear rates needed in the Fontan group. One explanation might be the increased hct with higher means in the Fontan group at T2, which might lead to increased cell-cell contacts, since AI and *y* at dIsc min were investigated in native blood samples (Connes et al., 2013). Therefore, Fontan patients might pay even more attention on keeping hydration recommendations during exercise to attenuate hct increase and alleviate aggregation-disaggregation balance.

Previous studies also reported inconsistent RBC deformability adaptations to acute exercise with results showing either no change, an increase or a decrease in RBC deformability (Yalcin et al., 2003; Suhr et al., 2012; Connes et al., 2013; Tomschi et al., 2018). Moreover, hypoxia is also discussed to affect deformability, but results are still controversial (Tuvia et al., 1992; Wei et al., 2016). Grau et al. (2016) demonstrated a decrease of deformability during severe hypoxia exposure, due to reduced RBC-NOS activity. This was explained by limited availability of oxygen, a co-factor for RBC-NOS. Also, Moon et al. (2016) found a decrease in deformability in hypoxia. The result of the present study did not indicate altered deformability or NO production during hypoxia or acute exercise in hypoxia in any of the groups. This is in concordance to the previously described findings, since the significant deformability decrease seen by Grau et al. and Moon et al. occurred at an altitude of 4,000 m asl. or higher and after long-term hypoxia exposure. At 2,500 m asl no significant differences were demonstrated. Therefore, altitude and exposition time to hypoxia might be too low respectively too short for deformability changes in the present study. Nevertheless, this is an important finding, since altitudes during leisure activities and flights correspond to the chosen altitude.

In summary, this study for the first time demonstrated increased RBC deformability in Fontan patients compared to matched controls. This finding might be related to elevated NO levels in the Fontan group, which might improve deformability. Also, increased EPO levels resulting in higher numbers of erythrocytes and increased hct might affect this finding. Higher NO production might be explained by increased (wall-)shear stress as a consequence of increased arterial stiffness. Differences in RBC aggregation were not found in native blood, what might indicate less aggregability.

## Conclusion

This study demonstrated that hypoxia exposure and exercise under hypoxia had similar impact on hemorheology in Fontan patients and controls and was clinically well tolerated. Nevertheless, aggregation-disaggregation balance was significantly increased in Fontan patients. Therefore, patients should consider regular hydration during exercise to avoid increased blood viscosity and thereby improve microcirculation/fluidity.

Briefly, there is no hemorheological indication for abstaining from short-term hypoxia exposure. Therefore, it might be save for Fontan patients. If long-term hypoxia exposure has similar impact on hemorheology and if it is still well tolerated by Fontan patients has to be proven.

Future studies should also focus on the reasons for increased NO expression and possibly altered aggregability of RBCs in Fontan patients.

## Data Availability Statement

The data that support the findings of this study are available on reasonable request from the corresponding author JH. The data are not publicly available due to restrictions described in the signed patient information letter ensuring that individual data will not be presented publicly in order to ensure the privacy of research participants.

## Ethics Statement

The study protocol was approved by the ethics committee of the University of Bonn (application number 335/2014) and corresponded with the Declaration of Helsinki. All participants/parents gave written informed consent to participate in this study.

## Author Contributions

JH, NM, UH, and JB were involved in the recruiting of patients, performed the exercise tests with the patients, and took the blood samples. DB and MG processed the blood samples. JH, DB, MG, and WB performed the lab work and discussed the results. MG, WB, JB, UH, and NM supervised the work. JH and MG processed the experimental data, performed the analysis, drafted the manuscript, and designed the figures. WB and DB aided in interpreting the results. All authors did experimental conception and commented on the manuscript.

## Conflict of Interest

The authors declare that the research was conducted in the absence of any commercial or financial relationships that could be construed as a potential conflict of interest.

## Abbreviations

AI: aggregation index
AIx: augmentation index
asl: above sea level
BGA: blood gas analysis
BMI: body-mass-index
eNOS: endothelial nitric oxide synthase
EPO: erythropoietin
Hb: hemoglobin
Hct: hematocrit
HLHS: hypoplastic left heart syndrome
LORRCA: laser-assisted optical rotational red cell analyzer
MCH: mean corpuscular hemoglobin
MCHC: mean corpuscular hemoglobin concentration
MCV: mean corpuscular volume
NO: nitric oxide
NYHA: New York Heart Association
PP: pulse pressure
PWV: pulse wave velocity
RBC: red blood cells
RBC-NOS: red blood cell nitric oxide synthase
RDW: red cell distribution width
ROS/RNS: reactive oxygen species/reactive nitrogen species
RR_dia_: diastolic blood pressure
RR_sys_: systolic blood pressure
sO_2_: oxygen saturation
SpO_2_: peripheral oxygen saturation
VEGF: vascular endothelial growth factor
$\dot{V}$ O_2(peak)_: (peak) oxygen uptake
*y* at dIsc min: minimum disaggregation shear rate.
